# Investigation of an Ultra Wideband Noise Sensor for Health Monitoring

**DOI:** 10.3390/s20041034

**Published:** 2020-02-14

**Authors:** Xuezhi Zeng, Joakim Robakowski, Mikael Persson, Albert Monteith, Andreas Fhager

**Affiliations:** 1 Department of Electrical Engineering, Chalmers University of Technology, SE412-96 Gothenburg, Sweden; 2 Department of Space, Earth and Environment, Chalmers University of Technology, SE412-96 Gothenburg, Sweden

**Keywords:** ultra wideband, time domain, microwave, biomedical applications, vital sign

## Abstract

Quick on-scene assessment and early intervention is the key to reduce the mortality of stroke and trauma patients, and it is highly desirable to develop ambulance-based diagnostic and monitoring devices in order to provide additional support to the medical personnel. We developed a compact and low cost ultra wideband noise sensor for medical diagnostics and vital sign monitoring in pre-hospital settings. In this work, we demonstrated the functionality of the sensor for respiration and heartbeat monitoring. In the test, metronome was used to manipulate the breathing pattern and the heartbeat rate reference was obtained with a commercial electrocardiogram (ECG) device. With seventeen tests performed for respiration rate detection, sixteen of them were successfully detected. The results also show that it is possible to detect the heartbeat rate accurately with the developed sensor.

## 1. Introduction

Pre-hospital care has experienced a remarkable evolution over the past decades and it has now become an integrated part of the healthcare system [1]. In the event of an accident or a sudden illness (e.g., stroke or trauma), medical personnel in the ambulance have to make decisions rapidly regarding the level of care, including which hospital the patient needs to be transported to, and whether to initiate some form of treatment. The objective is to reduce the risk for death and provide optimal care of the patient. The quick on-scene assessment and early intervention requires high level clinical reasoning and decision making skills. This not only poses high requirements on the clinician skills of the medical personnel but also places great demands on research and development of new methods and new technologies for assessment and treatment.

The patients, who are subject to these life and death decisions, would clearly benefit if medical personnel have access to the additional support of diagnostic tools. Besides, vital signs are also part of the pre-hospital assessment as they provide information about the abnormal physiological state of patient and thereby aid in the clinical evaluation of critically ill patients. The vital signs therefore help in establishing the evidence base for triage decisions [2] and emergency medical services quality management [3]. Due to the limitations of the established heartbeat recording technology, that is, electrocardiography (ECG), today only respiration rate was checked and recorded in the pre-hospital care in most of the countries. Report also shows that the emergency department triage nurses are poor at detecting abnormally fast and slow respiratory rates [4]. New technologies that could enable medical diagnostics in the pre-hospital settings and (or) aid in the reliable recording of vital signs are demanded.

Microwave technology has appeared as an outstanding candidate for pre-hospital medical care due to several favourable characteristics. The possibility to use a microwave-based technology in diagnostic situations relies on the existence of a contrast in the dielectric parameters between healthy and diseased tissue. At microwave frequencies the contrasts between these tissues are often at least an order of magnitude larger than for many other modalities [5,6]. This high contrast is what makes microwave frequencies interesting for medical applications. In addition, with the proper choice of the frequency, microwave signals can penetrate deeply into the skull and body, detecting abnormalities in tissues. As a candidate for vital sign detection, microwave technology is comfortable, safe and easy to use. Its operation is not restricted by the ambient brightness and the line of sight. Microwaves can penetrate the clothes and bedding and is therefore suitable for various monitoring situations. With the significant development in the telecommunication industry over the last decades, the size and cost of microwave electronic devices have been dramatically reduced, which enables the integration of microwave sensors in different environment.

The feasibility of using microwaves for stroke and trauma detection has been proved by clinical trials [7,8,9]. The detections of vital signs using both continuous wave (CW) [10,11,12,13] and ultra wideband (UWB) microwave radars [14,15,16,17,18,19] have also been investigated and showed very promising results. We therefore developed a microwave sensor dedicated for pre-hospital care with both the functionalities of medical diagnostics and vital sign monitoring. This work is dedicated to the investigation of the sensor for main vital sign monitoring, that is, respiration rate and heartbeat rate.

The paper is organised as follows—in Section 2, we introduced the sensor design, development and the signal processing technique. In Section 3, we described the measurement scenario and presented the results. In Section 4, we discussed the limitations of this work and proposed potential solutions.

## 2. Materials and Methods

Wideband microwave measurement is usually performed for medical diagnostics as it could provide more information about the body under investigation. The measurement could be done in either the frequency domain or the time domain, and the former is often very time consuming. In acute medical care, the measurement speed is crucial. We therefore chose to develop a time domain microwave sensor as it could potentially give a much higher measurement speed and simple architecture. In order to enable the diagnostics and health monitoring functionalities, several design requirements need to be met. In the following, the requirements on the sensor performance are discussed for the vital sign monitoring functionality.

### 2.1. Design Requirements

The chest wall displacement due to breathing and heartbeat is in the order of millimeter and sub-millimeter, respectively [20,21]. The detection of such small motion activities is challenging and sets high demands on the sensor performance.

#### 2.1.1. Dynamic Range

In a typical vital sign monitoring scenario, two antennas are placed very close to each other and side by side. One of the antennas transmits microwave signals to hit the body and the signal reflected back is captured by the other antenna. Besides the reflected signal, the received data also includes the direct coupling signal from the transmitting antenna and reflections from non-target objects.

With the assumption that there is only a single point scatter (i.e., the body) within the antennas beams, the received signal power of each frequency component, *f*, due to the body reflection in free space can be estimated from the following equation [22]:

(1) $$P_{r}\left(f\right)=\frac{P_{t}\left(f\right)G_{t}\left(f\right)G_{r}\left(f\right)\lambda^{2}\sigma \left(f\right)}{{\left(4\pi \right)}^{3}R_{t}^{2}R_{r}^{2}}.$$

Here $P_{d}$ is the transmitted power, $P_{r}$ is the received power, λ is the wavelength, $G_{t}$ is the gain of the transmitting antenna, $G_{r}$ is the gain of the receiving antenna, σ is the radar cross section of the body, $R_{t}$ and $R_{r}$ are the distance from the body to the transmitting antenna and the receiving antenna respectively.

For an ultra wideband time domain system, the received average power in time domain is calculated as:

(2) $$P_{R}=\frac{1}{T}\int |P_{r}\left(f\right){|}^{2}df,$$

where *T* is the period of the repetitively transmitted signal.

The overall intensity of the received signal is therefore dependent on the transmitted power, the antenna performance, the radar cross-section of the detected object and the detection distance. From the theoretical point of view, the vital detection reliability is limited by the noise floor of the system, which could be expressed as the following equation [23]:

(3) $$P_{N}=kT_{0}BF,$$

where $P_{N}$ is the noise power, *k* is the Boltzmann’s constant, $T_{0}=290$ K is the absolute temperature of the receiver input, *B* is the noise bandwidth and *F* is the noise figure of the system receiver.

The body-reflected signal needs to be higher than the noise floor in order to enable a detection and a low signal-to-noise ratio would result in inaccurate detection of the vital signs. Signal amplification is usually done at the transmitter or receiver side in order to improve the signal to noise ratio, but it is not always effective as it will also increase the intensity of the direct coupling signal, other scattered signal as well as the received noise. The direct coupling component from the transmitting antenna is usually much stronger than the body-reflected signal and a too high amplification would result in a “clipping” effect, causing the body-reflected signal distorted if these two signal components overlap with each other. We therefore need to design a sensor with a high input range and a low noise floor in order to achieve a high dynamic range.

#### 2.1.2. Timing Jitter

The position in which the transmitted signal hits the body varies slightly over the time due to the breathing and heartbeat activities. The reflected signals will therefore undergo different delays relative to the transmitted ones before being captured by the receiving antenna. In a time domain radar system, the detection of vital signs relies on the accurate measurement of these delayed signals. At rest, the variation of the chest wall displacement, caused by respiration, is between 4 and 12 mm [20] and the chest wall displacement due to heartbeat alone ranges from 0.2 to 0.5 mm [21]. These displacements corresponds to time delays within 27∼80 ps and 1.3∼3.3 ps respectively.

The signal measurement is affected by the timing jitter performance of the sensor, which includes both the sampling jitter and cycle to cycle jitter. The former leads the data samples taken at instants different from the intended regular sample points and the latter causes the time shift of the entire waveform. Due to the presence of these jitters, the actual sampled value of the $k^{th}$ sample for the $n^{th}$ waveform becomes:

(4) $$x\left(k,n\right)=x\left(nT+t_{c}+kT_{c}+t_{s}\right).$$

Here *T* has the same definition as in Equation (2), $t_{c}$ is the cycle-to-cycle jitter, $T_{c}$ is the sampling interval and $t_{s}$ is the sampling jitter. Both these effects will cause distortion on the measured signal and consequently affect the vital sign detection.

#### 2.1.3. Measurement Speed

**Figure 1:**
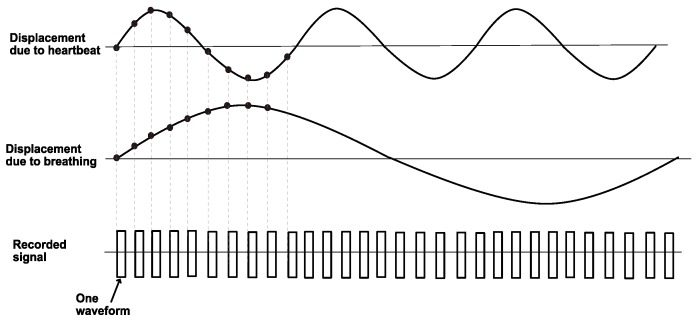
Signal recording in relation to the breathing and heartbeat rhythm.

Our chest expands and contracts periodically due to the respiration and heartbeat motion, and the key of the microwave-based vital sign detection is to detect the chest wall displacement, which could be modelled as a sinusoidal function, $m\sin(2\pi f_{d}t)$. Here *m* is the magnitude of the displacement and $f_{d}$ is the frequency of the periodical displacement, which reflects breathing or heartbeat rate [15]. With a time domain system, a UWB signal will be generated repetitively and transmitted to hit the body under test, and the body-reflected signals will be recorded successively, as shown in Figure 1, which illustrates the data recording in accordance with the breathing and heartbeat rhythm. The recorded signals will be used to obtain the displacement pattern and each recorded waveform corresponds to one sampling point on the displacement trace. In order to well reconstruct the displacement pattern, sufficient number of waveforms need to be collected within a certain time, which in turn puts a demand on the measurement speed. For example, if ten sampling points are used to reconstruct one period of the displacement pattern due to heartbeat (as shown in Figure 1), for a heartbeat rate of 120 beats per minute (BPM), the recording of one waveform should take less than t=1/[(120/60)·10]=50 ms.

### 2.2. Sensor Development

**Figure 2:**
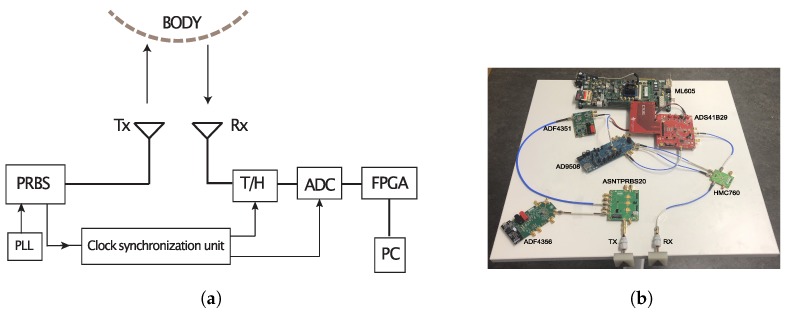
The block diagram of the developed sensor in Figure (**a**) and the measurement setup for vital sign monitoring in Figure (**b**).

Figure 2 shows the block diagram of the developed UWB noise sensor and the vital sign monitoring setup in our laboratory. The sensor works in the time domain, and it is composed of several off-the-shelf components. Each part of the sensors is described below in detail.

#### 2.2.1. Stimulus Signal and Signal Generation

The sensor uses a pseudo-random noise (PN) sequence as the stimulus signal. This type of signal could give a higher frequency domain signal to noise ratio (SNR) than a conventional ultra-short pulse [24] and is preferable for medical diagnostics where the signal usually undergoes strong attenuation. In addition, this type of signal is preferable from the signal generation point of view. An ultra short pulse is usually generated by using step recover diodes (SRDs) or nonlinear transmission lines (NLTLs) [25,26]. The SRD technique is simple and in-expensive, but the generated pulses have low amplitudes and a moderately high level of random jitter. Pulse generators built with custom designed NLTL generally have lower jitter, but are more complex in its design. A PN sequence, which is a sequence of binary numbers, for example, ±1, can be generated by a digital circuit and the generation is therefore simple and effective thanks to the rapid development of high-speed digital electronics. The PN sequence is generated by a pseudo-random binary sequence generator (PRBS) (ASNTPRBS20 from ADSANTEC). The generator is implemented with a 7-bit linear feedback shift register and the output is a maximum length sequence with the length of $2^{7}$-1 bits. The generator can be triggered by a clock varying from 20 MHz to 18 GHz and the obtained signal bandwidth can be up to 9 GHz (i.e., half of the triggering frequency).

**Figure 3:**
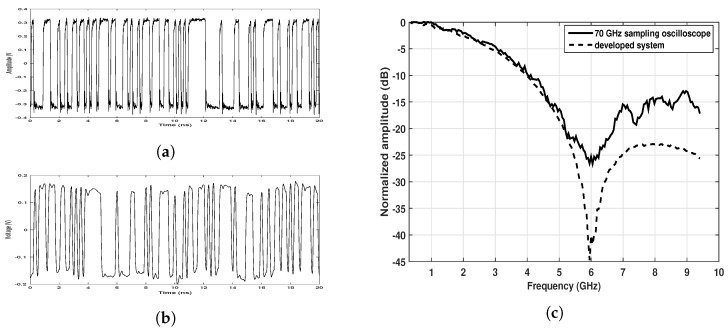
The sounding signal measured by (**a**) a 70 GHz sampling oscilloscope and (**b**) the receiver of the developed sensor, and (**c**) the corresponding frequency spectrums.

A 6 GHz clock generated by a phase-locked loop (PLL) synthesizer (ADF4356 from Analog Devices) was used to trigger the generator for signal generation. Figure 3a,b show the generated signal measured by a 70 GHz bandwidth sampling oscilloscope (86100C from Keysight Technologies) and the receiver of the sensor respectively. The former gives a very good estimate of the actual generated signal as the oscilloscope’s bandwidth is far beyond the signal’s bandwidth. The power spectra of the signals shown in Figure 3a,b are calculated and presented in Figure 3c for comparison. It can be seen that the signal measured with the developed sensor assembles the generated signal very well within 5 GHz, suggesting the ultra wideband charateristics.

#### 2.2.2. Data Acquisition

When hitting the body, the transmitted signal is partly reflected back and the reflected signal carries the vital signs of the body under investigation.

The real time acquisition of the reflected signal is challenging as it requires an analog to digital converter (ADC) with ultra-wide bandwidth and super high sampling rate. Although there are ADCs available on the market with the sampling rate of several Gsamples/s, their accuracy is a big concern due to the low resolution, high nonlinearity and high noise level. Moreover, these ADCs are extremely expensive.

In order to resolve these challenges, a wideband track and hold (T/H) (HMC760 from Analog Devices) was employed and placed ahead of a low-bandwidth ADC in order to relieve the bandwidth requirement on the ADC. The T/H has a 5 GHz input bandwidth and a superior jitter performance (<70 fs). The ADC (ADS41B29 from TEXAS INSTRUMENT) has a high speed (250 Msamples/s), high linearity and good noise performance. The acquired signal will first be sampled and held for a while by the T/H, and then the sampled value will be digitized by the ADC. An equivalent time sampling technique [27] was employed in order to obtain a sufficient effective sampling rate. The digital data will be resembled and recorded in a Field Program Gate Array (FPGA) board and then transferred to a PC.

#### 2.2.3. Clock and Timing

The generation of precise sampling clocks is crucial for the overall system performance. The clock and timing unit is composed of a fractional PLL synthesizer (ADF4351 from Analog Devices) and a clock distributor (AD9508 from Analog Devices) and its function is to generate the proper sampling clock signals. The reference clock input of this unit is given by the signal generator and this ensures the synchronization between the generated sampling clocks and the transmitted signals. The synthesizer allows precise and flexible tuning of the output clock frequency, which consequently can give any time resolution as demanded. The clock distributor has a phase offset function and the clocks triggering the T/H and the ADC were phased tuned in order to take into account the signal propagation delay from the T/H to the ADC.

Referring to the performance specifications of these components, the dynamic range of the sensor is about 55 dB and the total jitter is about 0.9 ps rms. The measurement time for one waveform is dependent on the number of sampling points and in this work, the measurement time is about 85 μs per waveform, which is far beyond the required measurement speed.

### 2.3. Signal Processing

The signal captured by the receiving antenna consists of several components. For the sake of simplicity, we assume that the antennas have isotropic radiation and they are ideally impedance matched to the sensor. If x(t) represents the transmitted signal, then the signal captured by the receiving antenna, *R*, can be expressed as:

(5) $$R\left(t\right)=P_{v}x\left[t-\tau \left(t\right)\right]+\overset{m}{\underset{i=1}{\sum }}P_{i}x\left(t-t_{i}\right).$$

Here $P_{v}$ is the amplitude of the body-reflected signal. $P_{i}$ is the amplitude for the $i^{th}$ unwanted echo. The time delay experienced by the body-reflected signal, τ, is a function of time, *t*, and it can be expressed as the following [15]:

(6) $$\tau \left(t\right)=2\left[d_{0}+d_{h}\sin\left(2\pi f_{h}t\right)+d_{r}\sin\left(2\pi f_{r}t\right)\right]/c_{0},$$

where $d_{0}$ is the distance between the body and the antennas, $c_{0}$ is the speed of light, $d_{h}$ and $d_{r}$ are the magnitudes of the chest wall displacement due to the heartbeat and respiration, $f_{h}$ and $f_{r}$ are the heartbeat and respiratory frequencies.

#### 2.3.1. Clutter Removal

The unwanted signals are referred to as clutter, and it mainly consists of direct coupling from the transmitting antenna and returns from background scatters. In a static environment, the clutter in each recorded waveform (refer to Figure 1) is the same while the body-reflected signal time shifted from waveform to waveform as the chest wall displacement due to the breathing and the heartbeat is quasi-periodic. Therefore, by averaging a large number of the collected waveforms, the body-reflected signal component will be suppressed and the resulting signal is a good estimate of the clutter [15]. We assume a neglected influence from environmental vibrations (e.g., building vibration).

Assume that *N* waveforms with the length *T* are recorded consecutively at the receiver and according to Equation (5), each waveform can be represented as the combination of the useful response and the clutter:

(7) $$R_{n}\left(k\right)=R_{v}\left(k\right)+R_{c}\left(k\right),$$

where k∈{1,…,K} is the $k^{th}$ sample and *K* is the number of the total samples of the waveform. $R_{n},n\in \left\{1,\ldots ,N\right\}$ is the $n^{th}$ recorded waveform, $R_{v}$ is the reflected signal from the body and $R_{c}$ represents the clutter.

The clutter is then estimated by averaging these *N* recorded waveforms. After the averaging, the first term on the right side of Equation (7) approaches zero and the remained part gives a good estimate of the clutter.

(8) $$\frac{1}{N}\overset{N}{\underset{n=1}{\sum }}R_{n}\left(k\right)\approx R_{c}\left(k\right).$$

By subtracting Equation (8) from Equation (7), the clutter free signal is obtained:

(9) $$R_{n}^{clutterfree}\left(k\right)=R_{n}\left(k\right)-\frac{1}{N}\overset{N}{\underset{n=1}{\sum }}R_{n}\left(k\right).$$

This signal is then further processed in order to extract the breathing rate and heartbeat rate.

#### 2.3.2. Slow Time Spectral Analysis

Slow time signal processing, which is commonly used in pulse radar, is employed here to extract the vital signs. Two time axes, fast time and slow time, are common concepts associated with radar signal processing [28]. Fast time represents the time running within each radar pulse and slow time is the time between the pulses.

We apply Equation (9) to the *N* recorded waveforms and put the obtained clutter free signals in an array as below:

$$M\left[N,K\right]=\left[\begin{array}{c} R_{1}^{clutterfree}\\ R_{2}^{clutterfree}\\ \vdots \\ R_{N-1}^{clutterfree}\\ R_{N}^{clutterfree}. \end{array}\right]$$

Each row of this array, M(n,1∼K)∀n∈{1,…,N}, is a fast-time signal and each column, M(1∼N,k)∀k∈{1,…,K} represents a slow-time signal. More explicitly, a fast time signal is a single waveform (refer to Figure 1) recorded at a certain moment, and a slow time signal is a data assembly from the clutter free signals at the same instant in relative to the beginning of the recorded waveforms. It has been theoretically demonstrated that the spectrum of a slow time signal consists of a train of delta functions centred at the frequencies of the harmonics and intermodulation products of the heartbeat frequency $f_{h}$ and respiratory frequency $f_{r}$ [15]. In order to identify these frequencies, fast Fourier transform (FFT) is performed on a slow time signal to obtain its frequency spectrum, *F*.

In order to improve the detection outcome, frequency spectrums of several slow time signals can be averaged according to the following equation:

(10) $$S=\frac{1}{N_{avg}}\overset{N_{avg}}{\underset{m=1}{\sum }}\frac{|F_{m}|}{\max\left(|F_{m}|\right)}.$$

Here, $F_{m}$ represents the frequency spectrum of the $m^{th}$ slow time signal. $N_{avg}$ represents the number of the averaged slow time spectrums. Each spectrum is self normalized in order to minimize the influence of any abrupt occurrence of strong interference.

#### 2.3.3. Bandpass Filter

Bandpass filters are applied to the entire frequency spectrum to separate the breathing signal and the heart beat signal. A Butterworth bandpass filter with a passband from 0.15 to 0.7 Hz (corresponding to a breathing rate of (9∼42 BPM) and stop band edge frequencies of 0.01 Hz and 0.9 Hz is used to obtain the respiration spectrum. The band-pass filter used for the extraction of heartbeat spectrum has a passband from 0.9 to 2.5 Hz (corresponding to a heartbeat rate of 54∼150 BPM) and the stop band edge frequencies are 0.8 Hz and 3.0 Hz. These frequency ranges should be able to cover the normal respiration rates and heart rates. The digital filters are designed to have a passband ripple of no more than 3 dB and a stop band attenuation of at least 20 dB. These bandpass filters are also expected to reduce the effect of the low frequency body movements, for example, the slow body motion when one is sitting or lying down.

## 3. Results

In order to test the methodology, a measurement was first made on a robot whose movement pace could be well controlled. Real human bodies were then tested. All the test were done in our lab with the developed sensor and a pair of patch antennas [29]. In order to avoid storing too much data, we create a time sequence in the FPGA to control the waveform recording. One waveform was recorded every 95 ms and in total 250 waveforms were collected. Each recorded waveform contained 3999 data points, resulting in a time resolution about 5.3 ps. In order to meet the speed requirement, we stored the data in the FPGA memory during the measurement and transferred the data to the PC after the whole measurement was completed. In order to increase the frequency resolution of the frequency spectrum, we zero-padded the slow time signal as described in Section 2.3.2 to extend the data points from 250 to 1000, resulting in a frequency resolution about 0.0105 Hz (0.63 BPM). This solution however will not improve the ability to resolve two closely spaced signals in the frequency spectrum as it does not contribute any additional information to the frequency content. In order to gain a better distinguishing ability, a longer data recording time is needed.

### 3.1. Detection of Robot Movement

**Figure 4:**
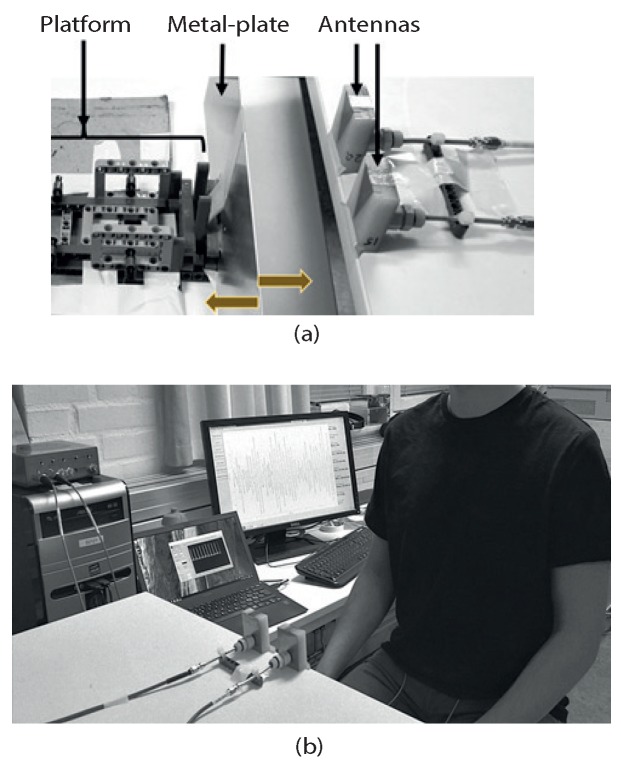
Measurement scenarios for (**a**) the robot movement detection and (**b**) the detection of human vital signs.

The measurement setup for the detection of the robot movement is shown in Figure 4a. The robot was placed close to the antennas and a flat metal plate was attached at the end of the robot arm in order to create a good reflecting plane. The motor drove the metal plate back and forth with the frequency of 8∼160 BPM.

**Figure 5:**
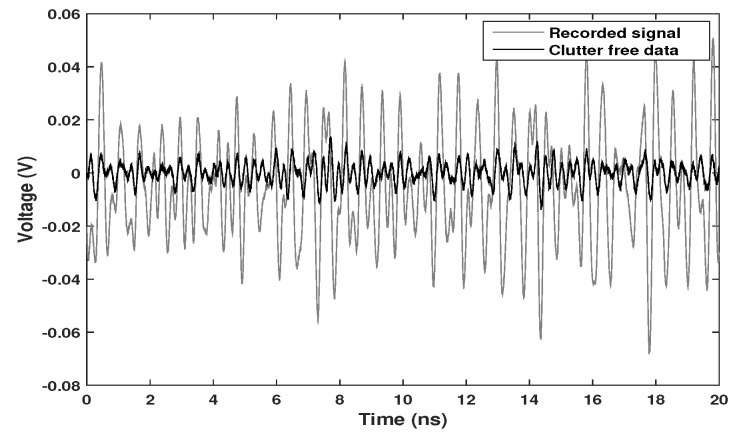
One waveform collected by the receiver and the signal after the clutter removal.

A test was made at the pace 18 BPM. Figure 5 shows one received signal before and after the clutter removal. The grey line represents the raw waveform, which corresponds to a single period transmitted PN sequence. The black line is the signal obtained after clutter removal.

**Figure 6:**
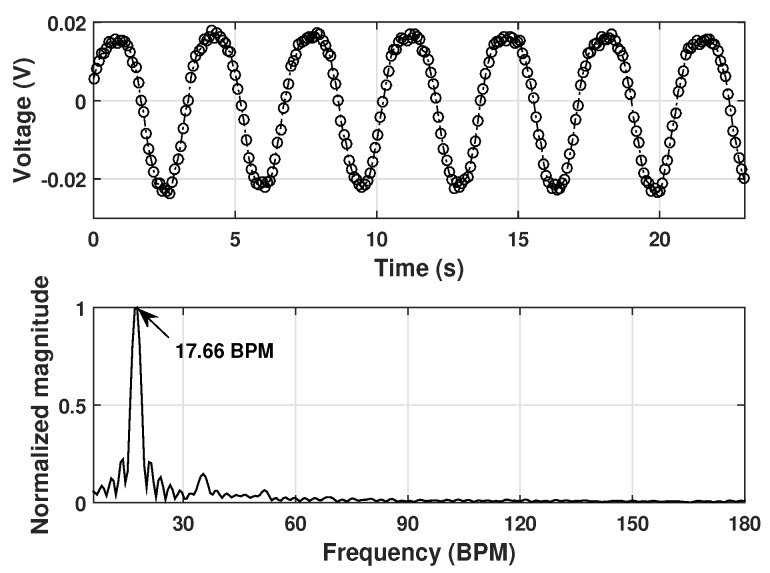
Detection results of the robot movement. The top figure shows a slow time signal and the bottom figure is its frequency spectrum.

The clutter free signals were then processed using the slow time spectral analysis. The top plot in Figure 6 shows one of the slow time signals and the bottom plot is the corresponding frequency spectrum. The result shows a peak at 17.66 ± 0.315 BPM, which is very close to the preset rate.

### 3.2. Detection of Respiration Rate

Tests were carried out on three different persons who were instructed to breathe at a certain rate following a software metronome installed in a smart phone. The person under test stood (or sat) at different distances to the antennas and in total seventeen tests were performed. Different distances and preset BPM values were considered in these tests. As the sensor was developed for acute medical care in the ambulances, it would be put very close to or even in contact with the patient. We therefore considered a detection distance within 50 cm.

**Figure 7:**
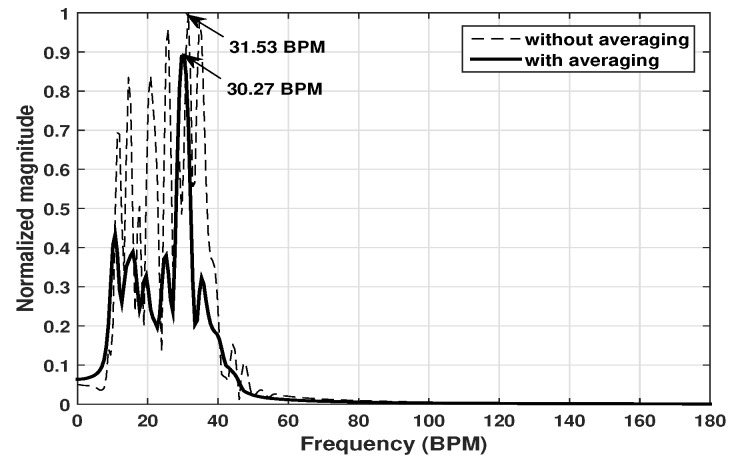
Respiratory frequency spectrum for the test no. 10.

The collected data were processed in the same way as for the robot movement detection. Figure 7 shows the obtained respiratory frequency spectra for one test example (test no. 10). In the example, the person stood facing to the antenna at a distance of 0.5 m, breathing at a speed of 30 BPM. Results obtained from one slow time signal and the averaging of $N_{avg}$ = 50 slow time signals (according to Equation (10)) were both presented. The result shows that there are several almost equally high peaks in the spectrum of the single slow time trace and it is thus difficult to make the detection decision. When more slow time traces were processed and averaged, the peak in the spectrum becomes much more dominant and the peak is located at 30.27 ± 0.315 BPM, suggesting a successful detection.

The detection results for all the test cases are summarised in Table 1. Breathing rates obtained without and with the averaging are both presented. The detection was considered to be successful if the difference between the detected rate and the preset value is within the resolution error (0.815 BPM), that is, half of the frequency resolution (0.315 BPM) plus half of the metronome resolution (0.5 BPM). The successful detections are highlighted in bold in the table.

The results show that when the detection was made using only one slow time signal, the successful rate was 10/17. When more slow time signals were used in the data processing, the detection rate becomes 16/17. There is a significant improvement on the detection outcome by using averaging. The improvement could be explained by the intrinsic characteristics of the employed waveform, as shown in Figure 3. As the chest wall displacement due to breathing and heartbeat is very small, the resulting slow time signal would be very weak if the received waveforms have a very low slew rate at the moment (e.g., around 0.5 ns in Figure 3a). As a consequence, it would be difficult to detect the vital signs especially when the noise is relatively strong. This could be solved by averaging many slow time traces corresponding to different moments.

### 3.3. Detection of Both Respiration Rate and Heart Rate

We investigated the possibility of detecting the respiration rate and heart rate simultaneously. A person under test stood or sat at a short distance from the antennas, as shown in Figure 4b. The person was breathing at a rate preset by the metronome, and an electrocardiogram (ECG) (Plessey PS25003 EPIC Demonstration Kit) device was used to acquire the heart rate as a reference.

The data collection and processing were the same as for previous test cases. The results were the average of $N_{avg}$ = 50 frequency spectrums.

**Figure 8:**
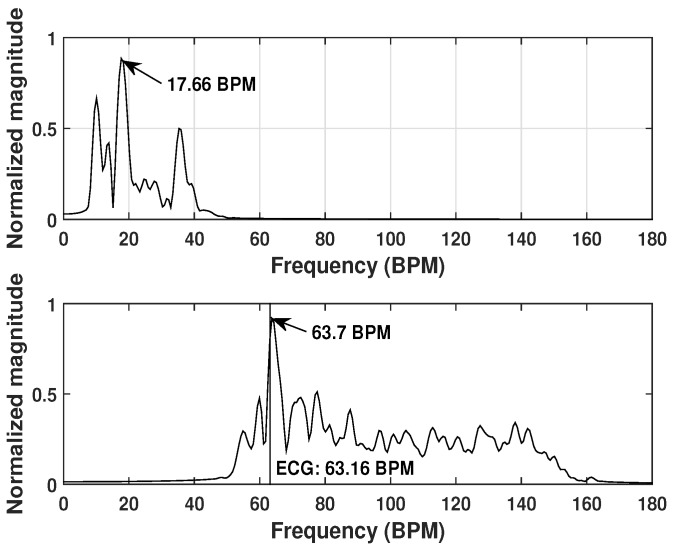
Respiration spectrum (**top**) and heartbeat spectrum (**bottom**) for test example 1.

Figure 8 shows the detection results for one test example. In this test, the person sat with his back towards the antennas at a distance of about 3 cm. The preset breathing rate is 18 BPM. Two bandpass filters were applied to the entire frequency spectrum in order to extract the respiration spectrum and heartbeat spectrum, respectively. The heart rate measured by the ECG device was presented in the figure as the vertical line. It can be seen that the highest peak in the respiration spectrum is located at 17.66 ± 0.315 BPM, indicating a successful detection of the breathing rate. The detected heart rate is 63.7 ± 0.315 BPM, which is very close to the ECG measured value (63.16 BPM).

**Figure 9:**
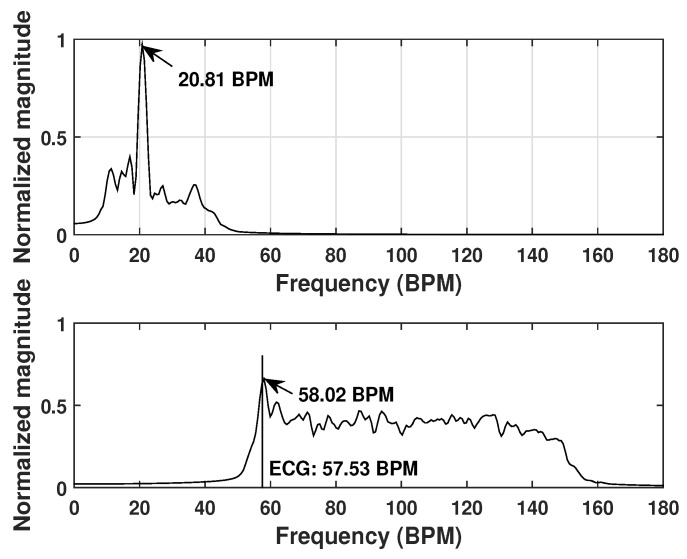
Respiration spectrum (**top**) and heartbeat spectrum (**bottom**) for test example 2.

Figure 9 shows the results for a test case where the breathing harmonics component is very close to the heartbeat frequency. In the test, the person sat facing towards the antenna and the preset respiration rate was 21 BPM. The respiration spectrum and heartbeat spectrum suggest the successful detection of both the respiration rate and the heart rate. The third harmonics of the breathing rate is presented as the secondary peak in the heartbeat spectrum and it is well differentiated from the heart rate frequency due to the high frequency resolution.

**Figure 10:**
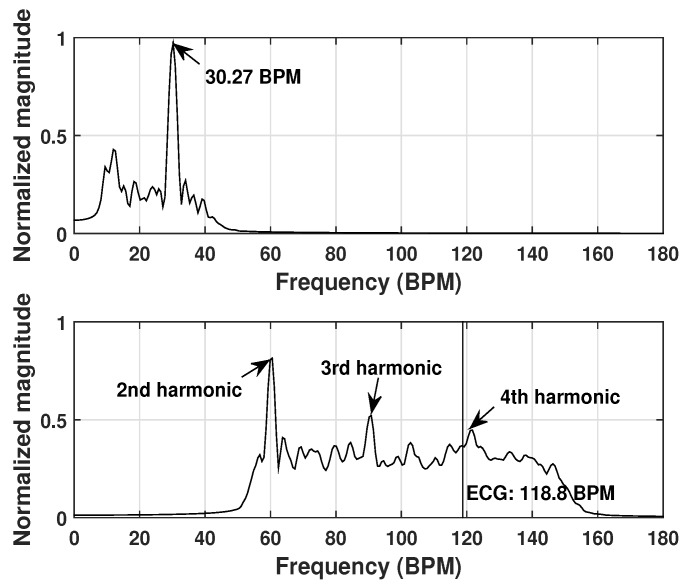
Respiration spectrum (**top**) and heartbeat spectrum (**bottom**) for test example 3.

It is however challenging to make the heart rate detection when the breathing harmonics are much stronger. Figure 10 shows the detection results of such a case. The person under test was measured after a few minutes exercising and in the measurement, the chest wall was in direct contact with the antenna. The detected breathing rate is 30.27 BPM and close to the preset rate 30 BPM in the metronome. In the heartbeat spectrum, the first, secondary and third peaks correspond to the second, third and fourth harmonics of the breathing rate respectively. The heartbeat frequency component is overwhelmed by the strong breathing harmonics, resulting in a missed detection. With an even better resolution and the use of a notch filter, this detection scenario should be able to resolved.

## 4. Discussion and Conclusions

We presented an UWB sensor dedicated to medical diagnostics and vital sign monitoring in pre-hospital care. The results demonstrated the functionality of the developed sensor for vital sign monitoring, and particularly, the detection of the respiration rate was highly reliable. A higher detection sensitivity could be expected if data averaging and filtering are used to reduce the effect of noise and jitter. As the sensor’s measurement speed is very high, data averaging is a very suitable way to improve the signal to noise ratio. In the future we will implement the hardware averaging in the FPGA.

There are however a few limitations which we need address in the future for practical use. One of them is that we assumed a negligible background vibration in this work. While this assumption is reasonable for the detection scenario in the laboratory where the building’s vibration is very weak, and the walls and windows are far away from the antennas, in real detection environment (e.g., an ambulance) the environmental vibration may cause problem if the vibration frequency lies in the respiration or heart rate spectrums. A well designed antenna with small beam could help in this situation as the reflections from other vibration sources will be much more reduced. In addition, an antenna with a broader band and smaller beamwidth is able to transmit and receive signals more effectively, which could potentially result in a more accurate detection. Another limitation is regarding the real time detection of the vital sign change. In this work, the recorded waveforms were firstly stored in the FPGA memory and the data transfer was done after all the 250 waveforms have been recorded. This solution results in a delayed detection of the respiration and heartbeat rate. In the future, the data processing will be directly done in the FPGA to achieve a real time detection and with more advanced processing techniques, such as time-frequency analysis based on wavelet transform, heart rate variability can also be obtained.

The idea is to use the sensor for trauma diagnostics and vital sign monitoring simultaneously. Although the primary use is in pre-hospital settings, it can also be used in trauma emergency wards, assisting in the post-treatment monitoring of the patients. We have previously demonstrated the high accuracy of the sensor for medical diagnostics [30], and a simultaneous use will be investigated in the future. An envisaged application example is to put an antenna belt around the chest wall, using the collected data to detect the chest injuries and monitor the vital signs. The measurement for diagnostic purposes will be completed within second, ensuring the minimal effect of the vital sign activities. The repetitively collected data in a longer time frame will then be used to monitor the patient’s vital signs.

The size of the sensor could be further reduced by using a more compact FPGA board without affecting the functionality and with additional effort, these off-the-shelf components can be integrated on a single small-sized PCB. The final goal is to have a sensor on chip with much reduced size and cost. As a low cost and automatic solution, it also allows for continuous vital sign monitoring in regular hospital wards for the early detection of patient deterioration.

## Figures and Tables

**Table 1:** The detected respiration rates for different test cases. The bold numbers indicate successful detections.

No.	Distance	Preset (BPM)	Detection (±0.315 BPM)	Averaged Detection (±0.315 BPM)
1	5–10	15	**15.14**	**15.14**
2	5–10	20	**19.55**	**19.55**
3	5–10	25	**25.23**	**25.23**
4	5–10	30	29.01	**29.64**
5	5–10	35	10.72	10.09
6	50	20	**20.18**	**20.18**
7	50	15	13.87	**14.51**
8	50	15	**15.14**	**15.14**
9	50	25	**25.23**	**25.23**
10	50	30	31.53	**30.27**
11	50	35	13.24	**34.69**
12	50	35	**35.32**	**35.32**
13	50 (sitting)	20	**20.18**	**20.18**
14	50 (sitting)	25	25.86	**25.23**
15	50 (sitting)	25	22.7	**25.23**
16	50 (sitting)	15	**15.14**	**15.14**
17	50 (sitting)	15	**15.77**	**15.14**
Success rate			10/17	16/17

## Abbreviations

The following abbreviations are used in this manuscript:

UWB	Ultra wideband
PN	Pseudo random noise
T/H	Track and hold
ADC	Analog to digital converter
PLL	Phase locked loop
BPM	Beat per minute
FPGA	Field Program Gate Array
ECG	electrocardiogram
